# “Are You a Boy or a Girl?”—A Missing Response Analysis

**DOI:** 10.3390/children10101695

**Published:** 2023-10-16

**Authors:** Andreas Heinz, András Költő, Ashley B. Taylor, Ace Chan

**Affiliations:** 1 Department of Health, IU International University of Applied Sciences, Juri-Gagarin Ring 152, 99084 Erfurt, Germany; 2 Health Promotion Research Centre, National University of Ireland, University Rd., H91 TK33 Galway, Ireland; andras.kolto@universityofgalway.ie; 3 Stigma and Resilience Among Vulnerable Youth Centre, University of British Columbia, Vancouver, BC V6T 1Z4, Canada

**Keywords:** gender, sex, non-response, survey methodology, LGBTQ, gender non-conforming

## Abstract

Many adolescent health surveys ask if respondents are male or female. Non-response may be due to fear of de-anonymisation or being a gender-nonconforming youth. The present study investigates the frequency of non-response and its potential reasons. To this end, data from 54,833 adolescents aged 11–18 from six countries, participating in the 2018 Health Behaviour in School-aged Children (HBSC) study, were analysed. Respondents were divided into three groups: (1) “Responders” who answered both questions on age and gender, (2) “Age non-responders” who did not answer the question on age, and (3) “Gender non-responders” who answered the question on age but not the one on gender. These groups were compared regarding their non-response to other questions and regarding their health. Overall, 98.0% were responders, 1.6% were age non-responders and 0.4% were gender non-responders. On average, age non-responders skipped more questions (4.2 out or 64) than gender non-responders (3.2) and responders (2.1). Gender non-responders reported more psychosomatic complaints, more frequent substance use and lower family support than responders. This study shows that age and gender non-responders differ in their response styles, suggesting different reasons for skipping the gender question. The health disparities found between the groups suggest that further research should use a more nuanced approach, informed by LGBT+ youth’s insights, to measure sex assigned at birth and gender identity.

## 1. Introduction

In many surveys, it is common to have a question on gender in the sociodemographic section of the questionnaire. Since its inception in 1983, the Health Behaviour in School-aged Children (HBSC), a World Health Organization collaborative cross-cultural study, measures gender using the question “Are you a boy or a girl?” [1]. Such a binary question can be problematic for two reasons. First, it does not distinguish between sex assigned at birth and self-identified gender, and second, it is not inclusive of those who would prefer using a gender descriptor other than boy or girl. To avoid choosing a response option that may not resonate with their gender identity, a possible reaction may be skipping the question. If subsequent analyses are broken down by gender, these respondents will be excluded, which means that their health risks will not be detected. The problem for research is that one cannot simply assume that all persons who skip the gender question belong to the group of gender-nonconforming persons because there may be other reasons to not answer this question.

This paper examines young people who did not answer the binary gender question in the 2018 HBSC study. To this end, three groups are compared: (1) gender non-responders who skipped the question about gender but answered the question about their age; (2) age non-responders who did not state their age, while it does not matter for the classification whether they indicated their gender; and (3) participants who answered both questions on gender and on age. The data suggest that there are different reasons behind the three (non)-response behaviours.

In the following, we outline which reasons for non-response are discussed in the literature and argue why a binary question about gender can be problematic for gender-nonconforming youth. We then provide some examples of health disparities that impact gender minority youth, and demonstrate how these affect gender-nonresponding youth in our sample, in contrast to their peers who answered the “Are you a boy or a girl” item.

### 1.1. Reasons for Item Non-Response

Various reasons for item non-response are discussed in methods research, but there is no overarching theory that covers all reasons for item non-response. Instead, item non-response is often explained by two theories that complement each other. The rational choice theory postulates that a question is not answered if a respondent perceives the costs of answering to be higher than the benefits [2]. Potential benefits of answering are that the question is interesting and that answering fulfils a social norm [3]. Among the costs of answering a question, various aspects are discussed and, here, the second theory comes into play, according to which answering a question is a cognitive process consisting of several steps [3,4,5]. The first step is understanding the question, i.e., respondents must first comprehend what the researchers want to know. This step can lead to item non-response, for example, if questions are ambiguous and respondents do not understand how the question is meant to be understood [5,6].

Once respondents have understood the question, the step of retrieval starts, i.e., respondents mentally access the information they need to answer the question. Item non-response can occur during this step if respondents do not have this information or if retrieving it is very difficult (e.g., retrospective questions on past events). The next step is judgement, i.e., respondents form a judgement based on the information retrieved. This step can also be cognitively demanding and lead to item non-response [7]. Subsequently, respondents will attempt to fit the answer into the given format. One reason for non-response can be that, from the respondents’ point of view, there are no suitable answer options for closed questions, i.e., the answer categories are not exhaustive. The final step of answering can also be influenced by the fact that a survey is a social situation. A source of non-response here could be that the question is sensitive, and giving an honest answer may be embarrassing for the respondent. Another source of non-response may be that respondents do not trust the anonymity of the survey and expect disadvantages from their answers [8,9,10]. This fear is known in surveys of organisations, such as small companies or school classes, where often little socio-demographic information is sufficient to identify individuals [11].

Item non-response can, therefore, occur in the case of ambiguous questions, cognitively demanding questions, non-exhaustive answer categories, sensitive questions, and questions that allow for de-anonymisation.

### 1.2. Binary Gender Questions in Health Surveys as a Potential Problem for Gender-Nonconforming Youth

Asking whether one is male or female is so common in surveys that, in face-to-face interviews, this question is often skipped by the interviewer because the answer seems obvious and they fill in the answer based on their perception of the interviewee, without asking them [12]. However, a question like “Are you male or female” can be problematic as it ignores the distinction between biological sex, which is expressed in the form of chromosomes, hormones and sexual organs, and social gender, which is a question of a person’s identity. Cognitive pretest interviews have shown that while sex and gender coincide (cisgender) for a large part of the population, for gender-nonconforming persons, the question can be difficult to answer [13]. Similarly, such a question may not be suitable for transgender and other gender-diverse (e.g., genderqueer or non-binary) persons [14]. In a few youth population health surveys, it has been estimated that around 0.2% to 2.7% of adolescents and young adults identified as transgender [15,16,17]. In recent investigations within the HBSC, it was found that 4.1% of adolescents in Germany aged 10–16 gave variant responses in terms of gender experience [18]. Among adolescents aged 15–18 in Spain, 1.1% identified as neither a boy or a girl, and 0.6% as other; percentage of all young people with non-cisgender identities was 2.2% [19]. These data show that a relatively small but remarkable proportion of young people might not easily answer the question “Are you a boy or a girl”.

- For transgender people, the question may be ambiguous, i.e., it is unclear whether the question is aimed at the sex assigned at birth or current gender identity [20].
- Since it is not clarified whether the item asks about biological sex or gender identity—and asking about being “a boy” or “a girl” is not being helpful in this sense—some participants may understand it as tapping into their sex. The dichotomous answers “male” and “female” ignore intersex persons, i.e., “people … born with sex characteristics (including genitals, gonads and chromosome patterns) that do not fit typical binary notions of male or female bodies” [21].
- For people who question their gender identity and people who reject a binary gender concept, a binary question may be inappropriate per se [22].

From the perspective of survey methodology, such a binary question is ambiguous because it seeks to measure two dimensions with one question. Moreover, it is not exhaustive because it does not comprise all characteristics that occur in reality. Thus, a question such as “Are you male or female” is particularly unsuitable for gender-nonconforming people, and numerous alternatives have been developed and tested, especially for surveys in these target groups [13,20,23]. However, a new standard to measure sex and gender that would be suitable for the adolescent population has not yet emerged.

It is necessary to measure gender and sex in a way that is both methodologically appropriate and accepted in the target group of the survey. This necessity is particularly relevant in health research as numerous studies have shown that gender minorities are disadvantaged in terms of health. A particularly affected group is adolescents, who consolidate their gender identity during this period [24].

### 1.3. Health Disparities of Transgender/Intersex/Gender-Nonconforming Adolescents

Research has shown a higher prevalence of certain health concerns among gender-nonconforming adolescents compared to their cisgender peers. In a study from Spain, cisgender youth aged 14–25 were compared with their transgender and non-binary peers. The latter groups more often reported verbal attacks at school and out of school, more psychological health problems, and suicidal ideation. In addition, they were less likely to report a high level of peer support and to feel happy [25]. The New Zealand Adolescent Health Survey found that transgender students and students who are unsure about their gender reported higher levels of depressive symptoms, self-harm, suicide attempts, weekly use of alcohol in the past month, school bullying victimisation and physical fight, as well as lower levels of peer support and feeling safe in the neighbourhood, compared to cisgender youth [15]. A survey of students aged 14–18 in Minnesota found that transgender and gender-nonconforming youth reported more risk behaviours, more emotional distress and mental health issues, and less protective factors [26]. A review of mental health of transgender youth based on fifteen articles confirms that they have higher rates of depression, suicidal ideation and behaviour, self-harm and eating disorders than their cisgender peers [27]. A landscape and research gap analysis of European studies on LGBTI+ youth demonstrated that the disproportionate burden of mental health issues in trans- and other gender minority youth is clearly linked to their identity-related minority stress and structural stigma [28].

Binary questions, such as “Are you a boy or a girl”, are obviously not suitable for measuring health aspects in gender minority groups. Rather, it is reasonable to assume that gender-nonconforming youth will not answer binary gender questions. However, this should not lead to the conclusion that all respondents who do not answer a binary question about gender belong to this group.

### 1.4. Research Questions and Hypotheses

For the reasons given above for item non-response, two fundamentally different constellations can be distinguished for not answering a question like “Are you male of female” or “Are you a boy or a girl”. First, for gender-nonconforming respondents, this question is problematic in itself. Those who question their gender identity may not know the answer. Transgender respondents may wonder whether the question refers to sex or gender. From the point of view of intersex respondents, the answers are not exhaustive, and for respondents who reject binary gender concepts, the question is entirely wrong.

For gender-conforming respondents, the question about gender is not a problem in itself, but some of them may fear that they can be identified with the combination of sociodemographic data. This fear may be very plausible, especially when examining school classes in a classroom setting.

To distinguish these two constellations, we used the question on age as the second socio-demographic indicator in addition to gender. Following the “Are you a boy or a girl” item, respondents were asked to provide the year and month of their birth. Their age was derived from these responses. Using the response behaviour to these two variables, we divided the respondents into three groups (Table 1).

“Responders” answered both sociodemographic questions and they represented the reference group. “Age non-responders” are those who did not answer the question on age, regardless of whether they answered the question on gender. This is based on the assumption that students who are afraid of being de-anonymised prefer to not state their exact age in a survey in a school class as a means of identification [11]. To skip only the question on gender, but to state their age, might not be sufficient to disguise their identity from their point of view.

“Gender non-responders” are those who answered the question on age, but not the one on gender. This behaviour can be expected in the case of gender-nonconforming persons for whom the question on gender is problematic in itself, whereas there is no reason for them to skip the question on age.

Based on the theoretical considerations, we aim to answer the following research questions and test two hypotheses using the presented classification of response behaviour:

**Research Question** **1:** *How many adolescents do not answer the items on gender compared to the question on age?*

This information helps to estimate how frequent both response behaviours are. The prevalence of gender non-response was compared with the prevalence of gender-nonconforming adolescents found in other studies. If the figures are similar, this may indicate that there are indeed many members of gender-nonconforming students among the gender non-responders.

**Research Question** **2:** *Does the prevalence of gender non-responders change with age?*

It is known from the methodological literature that non-response is generally lower in older children than in younger children because they are cognitively more capable of understanding and interpreting questions [29]. In the case of gender non-responders, however, we expect the prevalence to be higher among older students because research suggests that realising oneself to be gender-nonconforming is a long process, with the consolidation of one’s gender identity occurring after puberty [24,30].

**Hypothesis** **1:** *Age non-responders answer fewer questions in general compared to responders and gender non-responders.*

This hypothesis is based on the assumption that age non-response is due to the fear of de-anonymisation. If this were the main reason, then it could also be assumed that age non-responders would answer fewer questions in total in order to reveal less about themselves than gender non-responders. This generalised caution is not expected for gender non-responders, who, by definition, indicated their age. Apart from the question on gender, there is no obvious reason why they would answer fewer questions than other respondents.

**Hypothesis** **2:** *Gender non-responders report more negative psychosocial outcomes and health problems than responders.*

If the assumption is correct that the group of gender non-responders consists of many members of gender-nonconforming adolescents, then psychosocial and health problems that are typical for gender-nonconforming adolescents should be more frequent among gender non-responders. To be more precise, based on previous findings in the literature on gender-diverse young people, we assume that gender non-responders report the following more often than responders:

- More psychosomatic health complaints;
- Lower peer support;
- Lower family support;
- Lower classmate support;
- Lower life satisfaction;
- Higher percentage of bullying victimisation;
- Higher prevalence of smoking, drinking alcohol, drunkenness and cannabis use in the last 30 days;
- Higher percentage of poor self-rated health.

## 2. Materials and Methods

### 2.1. About HBSC

The HBSC is a World Health Organization collaborative cross-national study conducted every four years. In this study, we used data from the survey round in 2017/2018, carried out in 47 countries across Europe, Asia and North America. Cross-sectional data were gathered in school class settings based on nationally representative random cluster samples of 11-, 13- and 15-year-old adolescents in each participating country following a standardised international protocol. However, some countries covered a wider age range from 9 to 18 years old. More detailed information about the methodology of the HBSC study has been reported elsewhere [1].

For the present study, data from six countries were analysed to compare the response behaviour in different cultures. In addition, preliminary analyses showed that item non-response to questions on age and gender is rare, so a large number of respondents must be analysed in order to examine the hypotheses put forward. To maximise sample size and to investigate research question 2 (whether prevalence of gender non-response changes with age), we included all adolescents aged 11–18 in the analyses.

### 2.2. Measures

We used the following measures:

- *Gender:* To assess gender, the question “Are you a boy or a girl?” was asked.
- *Age* was measured by asking both year and month of birth. Those who did not answer one or both questions were considered age non-responders.
- *General item non-response*: The HBSC mandatory questionnaire used in 2018 consisted of 97 items (other than gender and month and year of birth). For 64 of these items, the number of questions not answered was counted. For the remaining 33 items, this was not possible, partly because they were coded 0/1 or due to skip patterns.
- *Psychosomatic health complaints*: The HBSC study surveys the prevalence of eight common symptoms in adolescents (e.g., backache and feeling low), which can be regarded as a measure of psychosomatic health [31]. Students were asked how often these complaints had occurred during the past 6 months, with answer options ranging from 1 “about every day” to 5 “rarely or never”. This study analysed how many complaints occurred more than once a week (i.e., options 1 and 2).
- *Peer support* and *family support* were measured using four items for each kind of support adapted from the Multidimensional Scale of Perceived Social Support [32]. These items (e.g., “My friends really try to help me” and “My family really tries to help me”) were measured using a seven-point Likert scale (1 = very strongly disagree, 7 = very strongly agree). This study used the mean of the four items, with higher values indicating higher peer support and family support, respectively.
- *Classmate support* was measured using the mean of three items developed by the HBSC study (e.g., “Other students accept me as I am”) asked on a five-point Likert scale. For the analysis, the original response scale (1 = strongly agree, 5 = strongly disagree) was reversed so that higher values represented more support from classmates.
- *Life satisfaction* was measured using the Cantril ladder [33]. This is an eleven-point measure of global satisfaction with one’s life, where 0 means “the worst possible life” and 10 is “the best possible life”.
- *Bullying victimisation*: Bullying was first defined in a preamble based on the definition of Olweus [34]. Then, students were asked two similar questions. The first question asked about the frequency of bullying victimisation at school, and the second one asked about cyberbullying victimisation in the past months. Answers ranged from 1 “I have not been bullied” to 5 “[I have been bullied] Several times a week”. Those who stated that they had been bullied and/or cyberbullied two times or more often (i.e., codes 3 to 5) were considered victims of frequent bullying.
- *Smoking, cannabis use, alcohol use* and *drunkenness* in the past 30 days were measured in a similar way using the following items: “On how many days (if any) have you smoked cigarettes?”, “On how many days (if any) have you used cannabis?”, “On how many days (if any) have you drunk alcohol?”, and “Have you ever had so much alcohol that you were really drunk?” All questions referred to the last 30 days, and the answer options ranged from 1 = “never” to 7 = “30 days or more” for smoking, cannabis and alcohol use and from 1 = “no, never” to 5 = “yes more than 10 times” for drunkenness. All answers greater than 1 were counted as smoking, cannabis use, alcohol use and drunkenness in the last 30 days, respectively. The measures for smoking, alcohol use, drunkenness and classmate support were developed and validated by the HBSC network [1].
- *Self-rated health* was measured using the item developed by Kaplan and Camacho [35]: “Would you say your health is…”, with response options of “excellent”, “good”, “fair” and ““poor”. Here, a bottom box count of the category “poor” was used.

Students who indicated to be younger than 11 or older than 18 were excluded from the analyses. Students who answered only half or fewer of the 64 questions (that were not 0/1 coded or required open-ended text responses) were excluded from the analyses. This was performed to avoid biasing the results by students with extreme non-response rates who, for example, did not answer the questionnaire because of time constraints or because they were highly unmotivated [7].

### 2.3. Statistical Analyses

All analyses were carried out using IBM SPSS Version 25 (Armonk, NY, USA). For dichotomous variables, percentage values were compared using Chi-square tests. Besides significance levels, Cramer’s *V* is indicated as a measure of effect size. For continuous variables, analysis of variance was conducted, and Games–Howell post hoc tests were used for pairwise comparisons of metric variables (general item non-response; number of psychosomatic health complaints; peer, family, and classmate support; life satisfaction). Omega-square (*ω*^2^) is given as the effect size for continuous variables [36].

## 3. Results

The total data set contained 61,513 participants, of which 5322 participants were excluded because they were younger than 11 and another 510 participants were excluded because they were older than 18. Furthermore, 848 participants were excluded because they answered half or fewer of the questions. Thus, the analytical data set comprised 54,833 students aged 11 to 18 years from Ireland, Luxembourg, Hungary, France, Scotland and the Dutch-speaking (Flemish) part of Belgium (Table 2).

Across all countries, 0.7% of the participants did not answer the gender question (Table 3). For the question on month of birth, non-response was slightly higher at 0.9%, and 1.0% did not indicate their year of birth. Cramer’s *V* shows that countries differ more in non-response rates for month and year of birth than for gender. For month of birth, non-response ranges from 0.3% in France to 3.1% in Scotland. For the question on gender, non-response ranges only from 0.4% (Hungary and France) to 1% (Luxembourg).

For the classification of non-response, answering and not answering the questions on age and gender were combined, as presented in Table 1. For all students who did not indicate their month or year of birth, the exact age could not be calculated, so they are referred to as age non-responders in the following. Across the sample, this applies to 1.6% of pupils, ranging from 0.6% in France to 3.5% in Scotland. Those students who did not indicate their gender, but did indicate their age, are referred to as gender non-responders in the following. At only 0.4%, this response pattern is very rare, and the differences between the countries are smaller than for the proportion of age non-responders, ranging from 0.3% in Hungary and Scotland to 0.7% in Flemish Belgium. The vast majority (98.0%) of students belong to the group of respondents, i.e., they answered both the question on gender and the questions on age.

**Figure 1:**
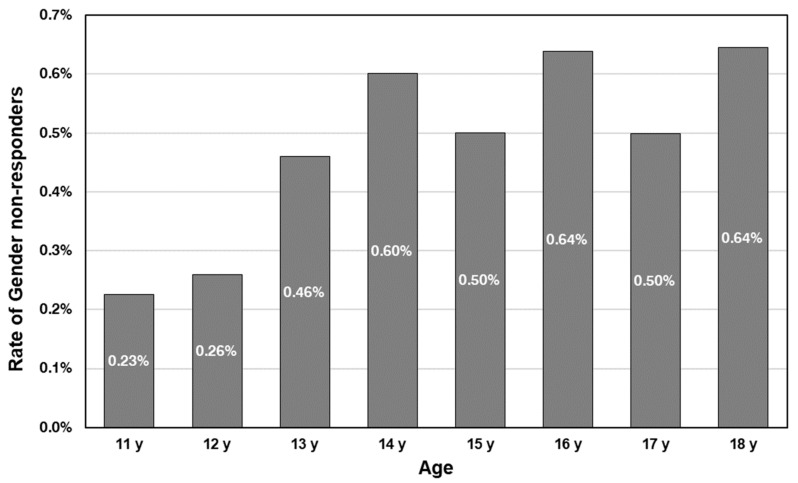
Rate of gender non-responders by age.

Gender non-responders, by definition, indicated their age, thus allowing us to report the frequency of this response pattern according to the age of the respondents (Figure 1). Of the respondents aged 11, only 0.2% were classified as gender non-responders. By the age of 14, this response behaviour becomes more frequent and remains at a level of 0.5% to below 0.7% until the age of 18. A comparison of column proportions showed that 11–12-year-olds were statistically significantly different from students aged 13–18. A logistic regression showed an association between age and being a gender non-responder. With each year, the odds of being a gender non-responder increases by 1.16 (95% confidence interval: 1.09–1.24).

**Figure 2:**
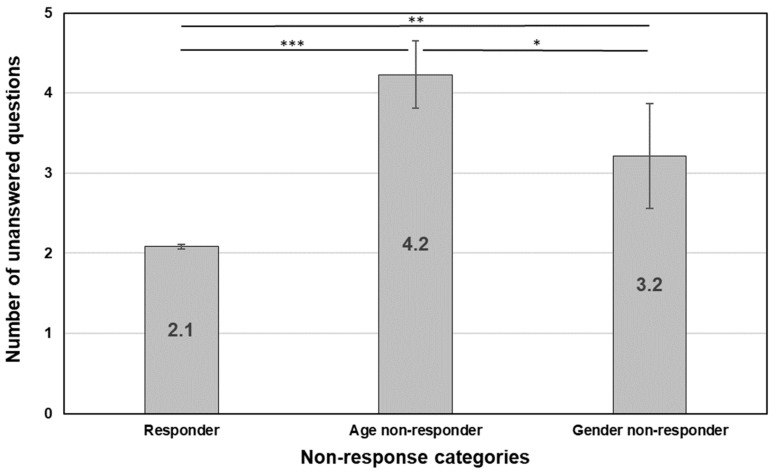
Number of unanswered questions by non-response classification (mean). * *p* < 0.05.; ** *p* < 0.01; *** *p* < 0.001. Error bars mark 95% confidence intervals.

On average, responders skipped 2.1 out of 64 questions (Figure 2), whereas age non-responders skipped twice as many (*p* < 0.001). Gender non-responders were in between as they skipped 3.2 items, with the difference being significant between responders and gender non-responders (*p* = 0.008) and between gender non-responders and age non-responders (*p* = 0.028). The difference across groups is significant: *F*(2) = 146.65, *p* < 0.001; but the effect size is low: *ω*^2^ = 0.005.

Table 4 shows the extent to which students differ from each other in terms of health, depending on their response pattern. The focus of the comparison is on responders and gender non-responders as we anticipated that gender non-responders have health concerns, which are more common for people who are not cisgender. On average, gender non-responders had 1.85 psychosomatic complaints that occurred more frequently than once a week. In the group of responders, it was only 1.48 complaints. Furthermore, gender non-responders felt less supported by their family than responders. However, responders and gender non-responders felt equally supported by their friends and classmates. The differences in life satisfaction were not statistically significant between these two groups.

As Table 5 displays, it was rare for students to rate their health status as “poor”, but this was over 2.5 times more common among gender non-responders than responders (4.7% versus 1.7%). The prevalence of substance use in the last 30 days was higher among gender non-responders than among responders. More than one third of gender non-responders had drunk alcohol in the past 30 days, but only about a quarter of responders had. Furthermore, 14.1% of gender non-responders reported drunkenness, but only 8.1% of responders did so. The difference is even greater in the use of cannabis. While only 6.3% of responders had used cannabis, more than twice as many gender non-responders reported the same. These are all significant differences. However, the difference in bullying victimisation is not significant.

## 4. Discussion

The binary question “Are you a boy or a girl” in adolescent health surveys is problematic for multiple reasons. First, it does not indicate whether it refers to biological (birth-registered) sex or gender identity. Second, the question makes it impossible for researchers to separate cisgender, transgender and non-binary adolescents. Third, the item and the binary responses exclude adolescents who would prefer other than binary answers.

In this study, we analysed patterns of non-response to better understand how the “Are you a boy or a girl” item works. Based on the previous theoretical literature, we assumed that gender and age responses are not missing at random, but there are two distinct patterns: (1) those who did not respond to the item on age (and subsequently, either did or did not respond to the item on gender), probably due to fear of de-anonymisation, and (2) those who responded to the item on age but subsequently skipped the item on gender—therefore, they may not fear that their identity will be revealed, but they may have had a problem with the way gender is asked.

First, we explored the proportion of the above two groups within the adolescent population. In general, 98% of the total sample answered the items on age and gender. However, not answering the item on gender is less common (0.7%) than skipping the question on month of birth (0.9%) or year of birth (1.0%). In addition, the range of item non-response rates across countries is much wider for the items on age (0.3 to 3.1%) than for the gender item (0.4% to 1.0%). These differences in prevalence suggest that there are different reasons for skipping these respective items. The rate of 0.7% of students who did not answer the gender question is a bit lower than the rate found in a pooled set of survey with Welsh young people aged 11–16 (1.3%) [37], as well as in the Minnesota Student Survey with participants in grades 9–11 (1.2%) [26]. However, our main group of interest is the group of adolescents who reported their age but not their gender (labelled “gender non-responders”), who represented 0.4% of our sample.

Our second research question is whether the prevalence of gender non-responders changes with age. We found a significant increase (odds ratio of 1.16 per age year); older adolescents were less likely to report their gender than their younger peers. We believe this is in line with non-conforming gender identity development. Previous evidence suggests that understanding and coming to terms with gender non-conformity is a long process, and gender-diverse identities tend to consolidate after puberty [24,30]. Thus, this age pattern indirectly suggests that older children may find the “Are you a boy or a girl” item increasingly problematic because, with age, the possibility increases that they do not identify with the terms “a boy” or “a girl” and would prefer using other gender descriptors.

Our first hypothesis is that age non-responders would answer fewer questions than responders and gender non-responders. We found statistically significant differences between all three groups, which is in line with our anticipation. Responders, on average, skipped 2.1 questions; age non-responders did not answer 4.2 questions; and gender non-responders skipped 3.2 questions. We assumed that age non-responders either fear de-anonymisation or have a general lack of interest and motivation to complete the survey, but this was not expected for gender non-responders, who, by definition, gave their age. For the latter group, we did not anticipate them to answer fewer items than responders. If age non-response is due to fear of de-anonymisation, we might assume that in contrast to gender non-responders and responders, they would generally be more hesitant to share other pieces of information about themselves than the other two groups.

This leads to our second hypothesis that gender non-responders would report poorer psychosocial outcomes and more health problems than responders. The pattern of the findings partly supported and partly contradicted this hypothesis. Gender non-responders reported significantly more health complaints and less family support, were more likely to rate their health as poor, and were more likely to show all four substance use behaviours (alcohol consumption, drunkenness, smoking and cannabis use in the last 30 days) than responders. If our assumption that gender non-responders skipped the item because it did not suit their gender experience is true, these results can be interpreted in line with the literature on the health of gender-diverse young people. For instance, transgender and other gender minority youth have higher odds than their cisgender peers to report substance use [16,38,39]. However, the health of gender minority young people, especially factors that boost and protect their well-being, remain a significant knowledge and evidence gap [28,40].

On the other hand, there was no statistical difference between the two groups in terms of classmate and friend support, life satisfaction or bullying victimisation. If we draw a parallel between gender non-response and gender minority identity, the latter result indirectly contradicts findings from the literature which unequivocally support transgender and other gender minority youth’s disproportionate bullying victimisation and mental health burden compared to their non-minority peers [16,25,26]. While we do not have a direct explanation for this divergence between our findings and those described in the literature, we have noted that gender non-responders represented a very small fraction (0.4%) of our sample. There is evidence that transgender children are more likely than their cisgender peers to be absent from school [38], and the reasons for this disparity (i.e., their fear of being bullied or ostracised) may also drive other gender-diverse youth to be absent. If all these youth were present in schools on the day of data collection, perhaps we would have seen a higher number of gender non-responders.

Our distinction between gender non-responders and age non-responders may not be substantiated. In other words, adolescents’ reasons for not responding to the items on gender and age may not differ across groups. However, the differences between the three groups in terms of non-response rates and some health outcomes, as well as the increase in gender non-response with age, do not support this notion.

Gender non-responders did not report different levels of peer and classmate support or life satisfaction than responders. If it were true that gender non-responders did not answer the “Are you a boy or a girl” item because they belong to gender minorities, based on the existing evidence, we should have seen that they reported less social support and less life satisfaction than responders. We speculate that those children who intentionally left the “Are you a boy or a girl” item (but not the items regarding their age) unanswered may be the ones who do not acquiesce to pre-set answers regarding their gender experience. They may be known to their friends and classmates as transgender or gender-diverse and may be supported by them, in contrast to gender-diverse kids who are not out to others and/or not being supported by their peers. This notion warrants further qualitative and mixed-method studies.

We believe that our study is strengthened by using nationally representative data from six European countries and the established methodology of the HBSC study. On the other hand, there are some limitations. Since we do not know the reasons for non-response, our explanations remain speculative. Apart from Central European Hungary, all other data are from Western European countries, while Eastern Europe is not represented at all. When assembling the data for this study, our colleagues from Eastern European countries (Ukraine, Lithuania, Latvia, Georgia, Estonia and Russia) found no or almost no gender non-responders in their raw datasets. This suggests that non-response on the “Are you a boy or a girl” item may also be due to cultural factors. The low numbers of gender and age non-responders limit the statistical power of the findings. Finally, gender minority young people are more likely than their cisgender peers to miss school due to fear of bullying and exclusion [38,41]; since our study was conducted in classrooms, there was no chance to reach out to students who were absent on the day of data collection.

One might ask why we did not further divide the group of age non-responders into a group that answered the gender question and a group that did not, as this way we “lost” some gender non-responders. After initial analyses and careful consideration, we decided not to do this mainly for the following reasons. First, we could not derive enough plausible reasons for these particular behaviours from the methodological literature. At most, one could argue that those who did not answer either question were particularly suspicious and wanted to avoid de-anonymisation at all costs, which might, of course, include gender non-conforming respondents. However, it would have been difficult for us to test this assumption in a meaningful way. We knew from our preliminary analyses that non-response rates are low and the respective groups would be very small if we tried all four combinations. As a result, the statistical tests would have had even smaller power. Even if we had found differences between the two subgroups of age non-responders, we felt we could not have offered a convincing explanation for them.

Second, the focus of our work is on gender non-responders because we suspect that these respondents are more likely to be gender-nonconforming students. For this reason, we did not pursue any further differentiation of age non-responders, especially as we could not formulate adequate theoretical justification for this division.

Our results partially support the notion that gender non-response in adolescent population health surveys may be linked to the formulation and response options of the item(s) classifying participants’ gender. The “Are you a boy or a girl” item, routinely used in HBSC since 1983, may not reflect the gender experience and identity of some young people, who, in return, may leave the item unanswered. Another challenge of the item is that it does not differentiate between biological sex and gender identity. Based on recommendations in the literature [42] and initial experiences with the 2018 HBSC study conducted in Spain [19], our working group is currently adapting a more inclusive approach to measuring sex and gender in the international HBSC study [43]. We hope this will enable researchers of the HBSC and other adolescent health surveys to separate birth-registered sex and gender identity and give voice to gender-diverse young people on their gender experience.

## Figures and Tables

**Table 1:** Classification of response behaviour.

		Answered the Question “Are You a Boy or a Girl”?	
		Yes	No
Answered the question on age	Yes	Responder	Gender non-responder
	No	Age non-responder	

**Table 2:** Description of the sample.

	*n*	Age Range	Mean Age	SD
Belgium (Flemish)	9551	11–18	14.7	2.2
Ireland	11,499	11–18	14.1	1.9
Luxembourg	8936	11–18	14.8	2.2
Hungary	4792	11–18	13.9	1.8
France	14,780	11–18	13.5	1.4
Scotland	5275	11–18	13.6	1.6
**Total**	54,833	11–18	14.1	1.9

**Table 3:** Item non-response (gender and age) by country.

	Item Non-Response Rate % (*n*)			Non-Response Classification		
Country	Month of Birth	Year of Birth	Gender	Gender Non-Responder (Did Not State Gender, but Age)	Age Non-Responder (Did Not State Age)	Responder (Stated Both Age and Gender)
Belgium (Fl.)	0.5% (45)	0.6% (58)	0.9% (85)	0.7% (65)	1.0% (93)	98.3% (9393)
Ireland	0.5% (63)	0.7% (84)	0.8% (92)	0.4% (41)	0.8% (92)	98.8% (11,366)
Luxembourg	1.1% (95)	2.6% (228)	1.0% (90)	0.6% (52)	3.1% (274)	96.3% (8610)
Hungary	2.3% (111)	0.4% (21)	0.4% (18)	0.3% (14)	2.4% (117)	97.3% (4661)
France	0.3% (40)	0.4% (59)	0.4% (55)	0.4% (52)	0.6% (91)	99.0% (14,637)
Scotland	3.1% (163)	2.3% (120)	0.5% (26)	0.3% (16)	3.5% (185)	96.2% (5074)
**Total**	0.9% (517)	1.0% (570)	0.7% (366)	0.4% (240)	1.6% (852)	98.0% (53,741)
	*χ*^2^ (5) = 472.04, *p* < 0.001; Cramer’s *V* = 0.093	*χ*^2^ (5) = 380.61, *p* < 0.001; Cramer’s *V* = 0.083	*χ*^2^ (5) = 53.76, *p* < 0.001; Cramer’s *V* = 0.031	*χ*^2^ (10) = 462.72; *p* < 0.001; Cramer’s *V* = 0.065		

**Table 4:** Health-related problems by non-response classification (continuous outcomes).

	R	GNR	ANR	R-GNR	R-ANR	ANR-GNR
Continuous Outcomes ^1^	M (SD)	M (SD)	M (SD)			
Number of health complaints (more than once a week; range 0–8)	1.48 (1.81)	1.85 (2.14)	1.57 (1.98)	*	n.s.	n.s.
Family support (1 = low, 7 = high)	5.63 (1.67)	5.19 (1.87)	5.41 (1.88)	**	n.s.	n.s.
Peer support (1 = low, 7 = high)	5.49 (1.67)	5.08 (1.91)	5.32 (1.78)	n.s.	n.s.	n.s.
Student/classmate support (1 = low, 5 = high)	3.88 (0.80)	3.82 (0.92)	3.76 (0.87)	n.s.	***	n.s.
Life satisfaction (0 = low, 10 = high)	7.56 (1.78)	7.29 (2.25)	7.35 (2.04)	n.s.	**	n.s.

^1^ The *p*-values in pairwise post hoc comparisons are adjusted using the Holm–Bonferroni method. * *p* < 0.05.; ** *p* < 0.01; *** *p* < 0.001; n.s. = not significant. R = responder (responded to both age and gender items). GNR = gender non-responder (responded to the age item but not the gender item). ANR = age non-responder (did not respond to the item on age and did or did not respond to the item on gender).

**Table 5:** Health-related problems by non-response classification (dichotomous outcomes).

	R	GNR	ANR	R-GNR	R-ANR	ANR-GNR
Dichotomous Outcomes (Comparison of Column Proportions) ^1^	% (*n*)	% (*n*)	% (*n*)			
Self-rated health “poor”	1.7% (928)	4.7% (11)	3.9% (32)	*	*	n.s.
Bullying victimisation	9.7% (5042)	13.0% (30)	12.1% (97)	n.s.	n.s.	n.s.
Drank alcohol last 30 days	25.9% (13,186)	35.6% (79)	34.9% (274)	*	*	n.s.
Drunkenness last 30 days	8.1% (4169)	14.1% (31)	15.6% (121)	*	*	n.s.
Smoked last 30 days	8.4% (4196)	15.0% (34)	19.4% (145)	*	*	n.s.
Cannabis use last 30 days	6.3% (2144)	14.9% (27)	16.8% (83)	*	*	n.s.

^1^ The *p*-values in the comparison of column proportions are adjusted using the Bonferroni method. * *p* < 0.05. n.s. = not significant. R = responder (responded to both age and gender items). GNR = gender non-responder (responded to the age item but not the gender item). ANR = age non-responder (did not respond to the item on age and did or did not respond to the item on gender).

## Data Availability

The data used in this study are available at a publicly available repository at https://www.uib.no/en/hbscdata/113290/open-access (accessed on 11 October 2023).
